# Association of Endocrine Therapy With Overall Survival in Women With Small, Hormone Receptor–Positive, *ERBB2*-Negative Breast Cancer

**DOI:** 10.1001/jamanetworkopen.2020.13973

**Published:** 2020-08-24

**Authors:** Sung Jun Ma, Oluwadamilola T. Oladeru, Anurag K. Singh

**Affiliations:** 1 Department of Radiation Medicine, Roswell Park Comprehensive Cancer Center, Buffalo, New York; 2 Department of Radiation Oncology, Massachusetts General Hospital, Boston

## Abstract

This cohort study examines the association of overall survival with endocrine therapy to treat hormone receptor–positive, *ERBB2*-negative breast cancer.

## Introduction

With routine screening mammography, nearly 1 in 5 breast cancer cases are invasive tumors smaller than 1 cm.^1^ However, for hormone receptor (HR)–positive, *ERBB2 *(previously *HER2* or *HER2/neu*)–negative, node-negative breast cancer, pT1a tumors (ie, tumors measuring more than 0.1 cm but not more than 0.5 cm in greatest dimension) were underrepresented in prior prospective trials, ranging from 6% to 13% of participants in prior trials.^2,3^ In the absence of strong prospective evidence, the National Comprehensive Cancer Network recommends consideration of adjuvant endocrine therapy for patients with pT1aN0 breast cancer without the routine use of multigene assay.^4^ Thus, we sought to examine the association of overall survival (OS) with endocrine therapy in this cohort of patients.

## Methods

In this cohort study, we queried the National Cancer Database query for women diagnosed with HR-positive, *ERBB2*-negative pT1aN0 breast cancer between January 2010 and December 2015 who were treated with or without adjuvant endocrine therapy. Follow-up occurred until December 2016; analysis was performed from January 2020 to March 2020. Institutional review board approval was obtained from the Roswell Park Comprehensive Cancer Center; informed consent was not needed because data in the National Cancer Database are deidentified and freely available to approved Commission on Cancer–affiliated investigators. Our study follows the Strengthening the Reporting of Observational Studies in Epidemiology (STROBE) reporting guideline. The primary end point was overall OS. Kaplan-Meier method, Cox multivariable analysis, and interaction analysis were performed for survival outcomes. To address potential confounders, the logit of propensity score matched all variables in a 1:1 ratio without any replacement. The standardized mean difference of variables was less than 0.1. To address immortal time bias, reanalysis was performed after excluding those who survived less than 6 months as a conditional landmark. Sensitivity analysis was also performed on a subgroup of patients who refused endocrine therapy. All analyses were performed with R version 3.6.1 (R Project for Statistical Computing). Statistical significance was set at 2-sided *P* < .05 (eAppendix in the Supplement).

## Results

A total of 42 708 patients, comprising 36 985 (86.6%) White patients and with a median (interquartile range [IQR]) age of 63 (54-71) years, met our criteria, including 31 509 patients (73.8%) and 11 199 patients (26.2%) with and without endocrine therapy, respectively. The median (IQR) follow up was 42.1 (24.2-62.1) months. On Cox multivariable analysis (adjusted for facility type, facility volume, age, race, income, insurance, Charlson-Deyo comorbidity score, year of diagnosis, surgery, radiation, number of lymph nodes examined, and hospital readmission), the receipt of adjuvant endocrine therapy was associated with improved OS (hazard ratio [HR], 0.69; 95% CI, 0.63-0.76; *P* < .001). The Table describes 7544 matched pairs, in whom similarly improved OS was observed (HR, 0.76; 95% CI, 0.66-0.88; *P* < .001) (Figure). There was no statistically significant interaction of endocrine therapy with other baseline characteristics, such as age, comorbidity score, or tumor grade. After excluding 1659 patients (3.9%) with survival of less than 6 months, the addition of adjuvant endocrine therapy remained associated with improved OS (HR, 0.74; 95% CI, 0.67-0.81; *P* < .001). Of 11 199 patients treated without endocrine therapy, 3492 patients (31.2%) recommended therapy by a clinician refused the treatment. Compared with those who received endocrine therapy, similar findings were observed among those refusing the treatment favoring endocrine therapy (HR, 0.80; 95% CI, 0.69-0.94; *P* = .007).

**Table.** Baseline Characteristics for Matched Cohorts

Characteristic	Patients, No. (%)		Standardized mean difference
	No endocrine therapy (n = 7544)	Endocrine therapy (n = 7544)	
Facility volume			
Low	358 (4.7)	359 (4.8)	.001
Intermediate	1542 (20.4)	1544 (20.5)	
High	5644 (74.8)	5641 (74.8)	
Facility type			
Nonacademic	5325 (70.6)	5325 (70.6)	<.001
Academic	2123 (28.1)	2123 (28.1)	
Not available	96 (1.3)	96 (1.3)	
Age, y			
<50	974 (12.9)	973 (12.9)	<.001
50-74	5057 (67.0)	5057 (67.0)	
≥75	1513 (20.1)	1514 (20.1)	
Charlson-Deyo comorbidity score			
0	6673 (88.5)	6671 (88.4)	.001
1	758 (10.0)	759 (10.1)	
≥2	113 (1.5)	114 (1.5)	
Income, median (IQR)^a^			
≥$50 353	5528 (73.3)	5525 (73.2)	<.001
<$50 353	2014 (26.7)	2017 (26.7)	
Not available	2 (0.0)	2 (0.0)	
Insurance			
None	18 (0.2)	18 (0.2)	.003
Private	3638 (48.2)	3634 (48.2)	
Public	3871 (51.3)	3874 (51.4)	
Not available	17 (0.2)	18 (0.2)	
Histology			
Ductal or lobular	7165 (95.0)	7165 (95.0)	<.001
Others	379 (5.0)	379 (5.0)	
Grade			
Well differentiated	4150 (55.0)	4150 (55.0)	.002
Moderately differentiated	2838 (37.6)	2842 (37.7)	
Poorly differentiated	244 (3.2)	243 (3.2)	
Not available	312 (4.1)	309 (4.1)	
Race			
White	7068 (93.7)	7065 (93.7)	<.001
Black	288 (3.8)	288 (3.8)	
Others	178 (2.4)	181 (2.4)	
Not available	10 (0.1)	10 (0.1)	
Year			
2010-2012	3538 (46.9)	3531 (46.8)	.002
2013-2015	4006 (53.1)	4013 (53.2)	
Lymph nodes examined			
≤2	4542 (60.2)	4542 (60.2)	.004
>2	2992 (39.7)	2991 (39.6)	
Not available	10 (0.1)	11 (0.1)	
Hormone receptor			
ER+/PR+	6979 (92.5)	6977 (92.5)	.001
ER+ or PR+ only	565 (7.5)	567 (7.5)	
Surgery			
Lumpectomy	4610 (61.1)	4609 (61.1)	.001
Mastectomy	2934 (38.9)	2933 (38.9)	
Others	0	2 (0.0)	
Margin			
Negative	7498 (99.4)	7497 (99.4)	.02
Positive	45 (0.6)	44 (0.6)	
Not available	1 (0.0)	3 (0.0)	
Radiation			
None	4082 (54.1)	4083 (54.1)	.001
External beam	3024 (40.1)	3025 (40.1)	
Others	438 (5.8)	436 (5.8)	
Radiation dose, median (IQR) Gy	60.0 (48.1-62.6)	60.0 (50.4-62.0)	.008
Readmission within 30 d			
None	7466 (99.0)	7465 (99.0)	.003
Unplanned	21 (0.3)	21 (0.3)	
Planned	43 (0.6)	43 (0.6)	
Others	2 (0.0)	2 (0.0)	
Not available	12 (0.2)	13 (0.2)	
Postoperative inpatient duration, d			
<1	4341 (57.5)	4445 (58.9)	.01
≥1	2498 (33.1)	2414 (32.0)	
Not available	705 (9.3)	685 (9.1)	

Abbreviations: ER, estrogen receptor; IQR, interquartile range; PR, progesterone receptor.

^a^ Median income based on data from the 2016 American Community Survey.

**Figure.**
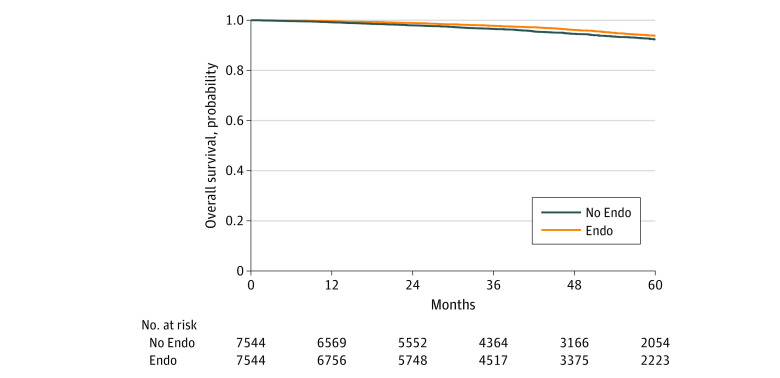
Kaplan-Meier Survival Curve After Matching Endo indicates endocrine therapy.

## Discussion

To our knowledge, this is the largest cohort study to evaluate the OS outcome of endocrine therapy for pT1aN0 breast cancer using a national registry database. The survival benefit identified in our study supports the National Comprehensive Cancer Network’s recommendation of adjuvant endocrine therapy in this cohort^4^ and is consistent with favorable outcomes previously described.^5^ However, the benefit of adjuvant endocrine therapy should be weighed against potential long-term toxic effects in select patients.^6^ Limitations of our study include unavailable variables such as performance status, toxicity profiles, and tumor recurrences, which may lead to unmeasured confounding despite matching. Reanalysis using a cohort of patients who declined recommended endocrine therapy revealed consistent findings. The risk of relapse from very small tumors remains significant enough to warrant adjuvant therapy. Our study further affirms clinicians’ decisions to recommend adjuvant endocrine therapy in this cohort of patients.

## Notes

Supplement. **eAppendix.** Supplemental Methods
