# Supplementary material for: Association of Endocrine Therapy With Overall Survival in Women With Small, Hormone Receptor–Positive, ERBB2-Negative Breast Cancer
Source: JAMA Netw Open. 2020 Aug 24;3(8):e2013973. doi: 10.1001/jamanetworkopen.2020.13973 (PMC7445593; doi:10.1001/jamanetworkopen.2020.13973)
Supplement: Supplement. — eAppendix. Supplemental Methods [file jamanetwopen-3-e2013973-s001.pdf]

Supplementary Online Content

Ma SJ, Oladeru OT, Singh AK. Association of endocrine therapy with overall survival in women with small, hormone receptor-positive, *ERBB2*-negative breast cancer. *JAMA Netw Open*. 2020;3(8):e2013973. doi:10.1001/jamanetworkopen.2020.13973

eAppendix. Supplemental Methods

This supplementary material has been provided by the authors to give readers additional information about their work.

© 2020 Ma SJ et al. *JAMA Network Open*.

eAppendix. Supplemental Methods

Our report is a retrospective observational cohort study using the National Cancer Database (NCDB).

All missing values were coded as unknown for analysis. Pertinent variables such as specific comorbidities, performance status, type and duration of chemotherapy, toxicity, tumor recurrence, and breast cancer specific mortality, were not captured in the NCDB. The primary endpoint was overall survival (OS) defined as the time interval between diagnosis and the last follow-up or death. Loss of patient to follow-up was the only censoring event.

Kaplan-Meier and log-rank tests were performed to analyze survival outcomes. Cox proportional hazard multivariable analysis (MVA) model was constructed using all statistically significant variables from the Cox univariable analysis followed by a backward stepwise elimination. Variables of interest include facility type, facility volume, age, race, Charlson-Deyo comorbidity score (CDS), income level, histology, grade, year of diagnosis, hormone receptor status, and types of treatments received.

The potential interaction between endocrine therapy and other baseline characteristics, such as age, CDS, and tumor grade, was performed by adding interaction terms to the Cox proportional hazard MVA final model.

Furthermore, to address the selection bias, propensity score matching was performed using each of the variables listed in Table 1. Some cases that were not matched based on baseline characteristics had to be excluded. Only those who received endocrine therapy were matched to those who did not receive endocrine therapy. Sensitivity analyses were performed using subgroup analyses. Such subgroups included those with survival of greater than 6 months to address immortal time bias as well as those who refused the endocrine therapy despite their clinicians' recommendations to adjust for potential unmeasured confounders.

All p values were two-sided and p values less than 0.05 were considered statistically significant.

© 2020 Ma SJ et al. *JAMA Network Open*.
